# A self-sustaining atomic magnetometer with *τ*^−1^ averaging property

**DOI:** 10.1038/srep28169

**Published:** 2016-06-30

**Authors:** C. Xu, S. G. Wang, Y. Y. Feng, L. Zhao, L. J. Wang

**Affiliations:** 1 Joint Institute for Measurement Science (JMI), Tsinghua University, Beijing 100084, China; 2 Department of Physics, Tsinghua University, Beijing 100084, China; 3 State Key Laboratory of Precision Measurement Technology and Instrument, Department of Precision Instruments, Tsinghua University, Beijing 100084, China; 4 School of physics, Beihang University, Beijing 100191, China

## Abstract

Quantum measurement using coherent superposition of intrinsic atomic states has the advantage of being absolute measurement and can form metrological standards. One example is the absolute measurement of magnetic field by monitoring the Larmor precession of atomic spins whilst another being the Ramsey type atomic clock. Yet, in almost all coherent quantum measurement, the precision is limited by the coherence time beyond which, the uncertainty decreases only as *τ*^−1/2^. Here we show that by non-destructively measuring the phase of the Larmor precession and regenerating the coherence via optical pumping, the self-sustaining Larmor precession signal can persist indefinitely. Consequently, the precision of the magnetometer increases with time following a much faster *τ*^−1^ rule. A mean sensitivity of 240 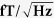 from 1 Hz to 10 Hz is realized, being close to the shot noise level. This method of coherence regeneration may also find important applications in improving the performance of atomic clocks.

Magnetometers based on atomic spin have achieved great progress in recent years and magnetometers with different characteristics find important applications in various fields^1^,^2^,^3^. High sensitivity below 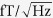 has been demonstrated by Kominis *et al*.^4^ with a potassium SERF (spin exchange relaxation-free) magnetometer and by Budker *et al*.^5^ with NMOR (nonlinear magneto-optical rotation) magnetometers, making atomic magnetometers comparable to or even surpassing the state-of-the-art SQUIDs (superconducting quantum interference devices)^6^,^7^. In the field of ultrasensitive magnetic field detection, measurement of an external magnetic field can be realized by monitoring the Larmor precession of atomic spins^8^. However, the atomic spin will inevitably suffer from decoherence and decay to thermal equilibrium after a lifetime *τ*_*atom*_, which limits the precision of the measurement. Hence, extending the coherence time is the key in improving the performance of all coherent quantum systems^9^,^10^. Here, we investigate and report a novel, self-sustaining atomic spin magnetometer based on coherent optical pumping. By synchronously switching on the pumping light at the Larmor precession frequency triggered by non-destructive measurement of its phase, the atomic spins can be regenerated coherently against the relaxation and thus maintain its precession indefinitely. The phase coherence time of the Larmor precession can thus be extended to a scale much longer than *τ*_*atom*_. Consequently, the self-sustaining magnetometer utilizes the phase information, instead of merely the frequency of the Larmor precession to measure the magnetic field and shows the attractive feature that its measurement uncertainty averages down in time at a faster 1/*τ* rate.

The self-sustaining atomic magnetometer has several other advantages. It can self-oscillate from noise without requiring any initial preparation. Via the gyromagnetic ratio, it converts the measurement of magnetic field to that of frequency and time – which has the highest precision among all physical standards. Thus, this magnetometer is an absolute measurement device and can form a metrological standard. We note that spin precession driven by periodically modulated optical pumping was first studied by Bell and Bloom^11^ where persistent spin polarization was obtained. In the present work, we measure the precession spin signal’s phase via non-destructive measurement instead of by tracing the atoms’ absorption^12^,^13^,^14^, minimizing disruption to the precession itself. Finally, the continuous oscillating signal makes it much more convenient to apply further signal processing in many applications. For example, phase-locking of the precession signal to a reference local oscillator can produce a highly sensitive error signal to lock the magnetic field. The phase-locking stabilization of magnetic field can be very helpful when an ultra-stable magnetic field is needed^8^.

The magnetometer monitors the Larmor precession of the electron spin of an atom in the magnetic field. For a standard free precession process, the spin is first polarized along the propagation direction of a circularly polarized pump light, then the pump light is turned off and the spin starts precession around the external field  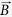 . With the presence of decoherence, for example, caused by collision or by the atoms moving out of the detection region, the average value of the spin will relax to zero after a certain lifetime *τ*_*atom*_. For a time interval *τ* > *τ*_*atom*_ the measurement can be repeated for a number of *τ/τ*_*atom*_ times, resulting in a sensitivity of^1^


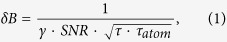


where *SNR* is the signal-to-noise ratio and *γ* is the gyromagnetic ratio. If the system is limited by the spin projection noise only, then *SNR* equals 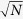 for an ensemble of *N* atoms. For a measurement time *τ* shorter than *τ*_*atom*_, let *τ*_*atom*_ equal to *τ*. Then, the sensitivity will improve as 1/*τ*. For a measurement time *τ* much longer than *τ*_*atom*_, the sensitivity improves as 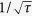 due to the uncorrelated phase in each repeated measurement of time interval *τ*_*atom*_. The polarization preparation by pumping in every measurement cycle destroys any phase coherence beyond time *τ*_*atom*_, thus resulting in the 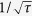 rule.

The spin self-sustaining method replenishes the atomic spin coherently. We illustrate the method using ^85^Rb atoms in the experiment as shown in Fig. 1(a). Setting the 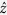 -axis along the magnetic field, at *t* = 0 a circularly polarized light along the 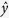 -axis pumps all the atoms into the magnetic sublevel state |*m*_*Fy*_ = 3>. This pump light orients all atomic spins along the 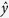-direction. Then the pump is turned off and the atomic spins precess and relax in dark. At time *t* = *2π/ω*_*L*_ the population will evolve back to the initial |*m*_*Fy*_ = 3> state. If the pumping light pulse is switched on right at this moment again, all atoms in |*m*_*Fy*_ = 3> will remain unaffected while atoms in all other states due to relaxation will be pumped back to |*m*_*Fy*_ = 3>. In this way, the spin is regenerated by the coherent pumping field and can maintain a very long lifetime.

The spin prepared via this method persists precession and behaves like a forced oscillator. The continuous signal can be described by 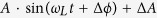, where Δ*A* and Δ*ϕ* represent the amplitude and phase noise, respectively. *ω*_*L*_ is the Larmor frequency. Owing to the spin self-sustaining method, the amplitude of the signal tends to be a constant value, reducing the amplitude noise. Hence, phase noise is the main factor that influences the measurement of 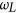. The uncertainty Δ*t* in the moment to turn on the pumping beam will mis-orient the spin with a phase error *ω*_*L*_Δ*t*, introducing noise to Δ*ϕ*. Since each pumping moment is determined from the phase information of the Larmor precession signal measured before that moment, the phase of the Larmor precession in this method behaves like a “self-referencing clock”^15^. The uncertainty in each pumping moment will cause phase error to accumulate. In the situation when this cumulative phase error is small, the spin coherence is maintained, resulting in a *τ*^*−*1^ reduction in magnetic field measurement uncertainty. When the accumulated phase error during a time interval *τ*_0_ is large enough to destroy the phase coherence, the measurement will then behave as 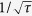. The *τ*^*−*1^ rule will change to a *τ*^*−*1/2^ slope at time *τ*_0_, as analyzed in detail in ref. 15.

## Results

**Figure 1:**
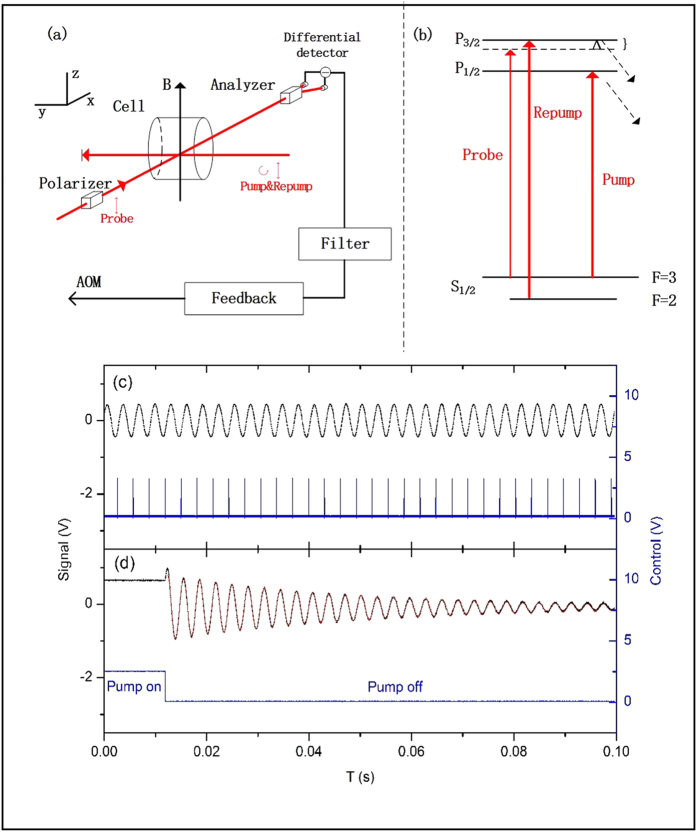
Schematic diagram of the experimental apparatus. (**a**) The circularly polarized laser and the linearly polarized laser serving as the pumping and repumping light, respectively, go through the same AOM (acousto-optic modulator) so that they can be switched on and off simultaneously. The probe laser propagates through the Glan-Taylor polarizer, the cell, and the Wollaston analyzer in sequence along 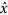 to form the Faraday rotation measurement. Its beam size is about 2 mm and its frequency is red-detuned by Δ∼4 GHz from *F* = 3 → *F*′ of the D2 line. The three lasers come from three independent, tunable external cavity diode lasers and they are not phase locked to each other. The 20 mm long, 20  mm in diameter cylindrical cell used here is a self-made α-olefin coated cell containing natural abundance rubidium atoms. The cell is placed inside a five-layer μ-metal magnetic shield casing with a shielding factor of better than 10^5^. A pair of Helmholtz coils controlled by a stable current source generates the uniform magnetic field *B* along 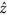 for spin precession. The experiment is performed at room temperature. The signal goes through a band-pass filter and then to the high speed zero-crossing comparator based on chip MAX999 (Maxim Integrated Products). The PCB of the comparator is carefully designed to reduce the high frequency electrical noise. For simplicity the magnetic shielding, AOM, and coils are not shown. **(b)** Energy levels of ^85^Rb atom. **(c)** Spin precession signals observed in self-sustaining setup, and **(d)** “single-pump free precession” setup. The black line is the precession signal, the blue line is the control signal and the red line is a sine-decay fitting giving a decay time constant of approximately 30 ms. The optical pumping is on when the control signal is high. The duration of the pumping pulse for spin self-sustaining method is 10 μs and the timing uncertainty of the rising edge in the trigger is measured to be 1 μs.

The configuration of the self-sustaining magnetometer is illustrated in Fig. 1(a,b). The signal of the probe light is proportional to the electron spin projection, <*S*_*x*_>, along its propagation direction^16^ 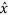. Due to the very large detuning set for the probe beam, it will only monitor the Larmor precession without directly influencing the atomic population in the states. This is a key feature of nondemolition measurement. We set the probe and pump beams in an orthogonal configuration such that when the spin precesses back along the pump light propagation direction, after one precession cycle, the signal of the probe just crosses the zero. This way, multiplicative amplitude error will be minimized. Coherent optical pumping of the spin is achieved by detecting the zero-crossing point with an analogue zero-crossing comparator and the rising edge of the output of the comparator is used as the trigger to turn on the pulsed optical pumping and repump light. This feedback loop synchronizes the pump with the Larmor precession. We’d like to point out that this synchronization technology can achieve high performance and synchronization of the rising edge by phase locking in a pure electrical system can be accurate to picosecond level.

Spin precession signals of the self-sustaining magnetometer are shown in Fig. 1(c,d). As shown from the signal observed in a “single-pump free precession” setup (d), the atomic spins have a lifetime of 30 ms, primarily limited by wall collisions. As shown in (c), the spin self-sustaining method keeps the atomic spins oscillating indefinitely.

**Figure 2:**
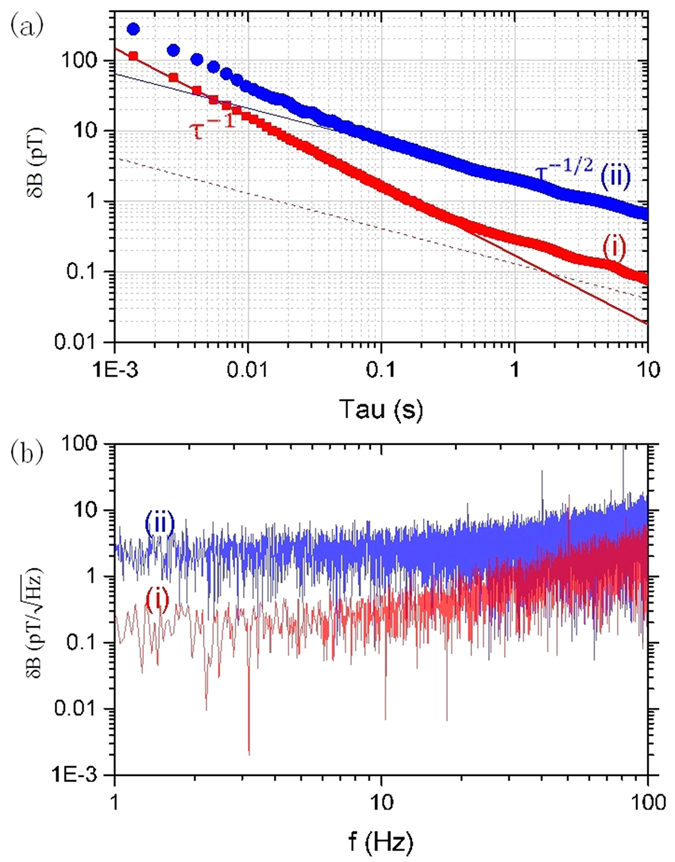
Allan deviation of the “free-precession repeated” and spin self-sustaining method. (**a**) Since *τ*_*atom*_ equals to 30 ms, we set a repeated pumping sequence as 10 ms pump followed by 30 ms free precession for the former method, according to Eq. (1). Other experiment conditions are the same for both methods. The read time for each data is equal to the inverse of the Larmor frequency *ω*_*L*_ = 2π · 700 H_Z_. In both cases, the Allan deviation is calculated with the data in 180 s and converted to the magnetic field sensitivity using the gyromagnetic ratio *γ*. The red (i) and blue (ii) solid line are the *τ*^−1^and *τ*^−1/2^ fittings, respectively. The red dash line represents the spin projection noise limit. **(b)** FFT of the Larmor frequency data showing the equivalent magnetic noise power density. The FFT spectrum is obtained from the first 20 s of recorded frequency data using the RMS method^21^. We find that the average noise level of 1 Hz to 10 Hz is 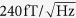 for the spin self-sustaining method, while being 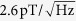 for the “free-precession repeated” method.

For comparison, we test the performance of the magnetometer in both the “free-precession repeated” mode and the spin self-sustaining method, as shown in Fig. 2. In order to minimize the influence of magnetic field fluctuation, we monitor the coils current *I* with a 6^1^/_2_ digital multimeter. The cross-correlation coefficient between the multimeter reading and the magnetometer measurement result is 0.94. Thus we can subtract the noise caused by the fluctuation in the coils’ current from the measured magnetic field *B*, through a linear relation 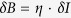, where *η* is the conversion coefficient in unit of Tesla/Ampere.

## Discussion

Timing errors may arise in the feedback loop. The possible noise comes from two parts: the noise in the Larmor precession signal itself and the one in the electronics. We need to reduce the intensity fluctuations in the probe light in the former part. In the latter part, one should use low noise photodetectors and filters. Great care must be taken to minimize the high frequency electronic noise in the high speed zero-crossing comparator since any noise in the comparator itself directly contributes to the timing error.

We analyze the sensitivity of our magnetometer by recording the continuous Larmor precession signal by a high speed 16-bit DAQ (data acquisition) card. The time sequence of zero-crossing points of the recorded signal is obtained with a linear fitting method near the zero point. The relative uncertainty in Δ*T/T*, with *T* being the period of Larmor precession of a single cycle, of each zero-crossing point due to data acquisition and processing is estimated to be below 5 × 10^−5^. Thus the main uncertainty in Δ*T/T* comes from the signal itself. Allan deviations^17^ shown in Fig. 2(a) display features clearly in accordance with the analysis. The self-sustaining method can retrieve magnetic field, *B,* from phase information of the Larmor precession. The phase noise Δ*ϕ* results in an uncertainty in *B* through 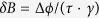. When the cumulative phase error due to pumping is small, Δ*ϕ* is almost constant with time, leading to the 1/*τ* slope. The 1/*τ* rule can extend to about 300 ms, during which, the spin Larmor precession maintains phase coherence; *τ*_*atom*_ is only around 30 ms. It is followed by then turning into a 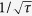 rule. The Allan deviation also shows the superiority of the spin self-sustaining method over the “free-precession repeated” mode on noise averaging property over time. The spin self-sustaining method can achieve a sensitivity of 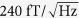, much reduced compared to the “free-precession repeated” method. The shot noise limit of spin projection according to Eq. (1) is estimated to be about 130 f T at 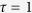 s for the experimental system.

The FFT (fast Fourier transform) spectrum, Fig. 2(b), supports the analysis above. The different slope in the 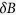 vs. 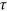 log plot in Fig. 2(a) corresponds to different noise types in the frequency domain with the form^18^ *S*_*y*_(*f)* ∝ *f*^*α*^. Here, *S*_*y*_(*f* ) represents the spectral density of the fractional frequency fluctuations *y* = Δ*f/f* and *α* is the power-law exponent that depends on the noise type. For magnetometers, the Larmor precession frequency is proportional to *B* through the gyromagnetic ratio and thus *S*_*y*_(*f* ) represents the spectral density of the relative magnetic field fluctuations. Since a 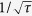 Allan deviation in time domain corresponds to white noise in the frequency domain with *α* = 0, the 1/*τ* Allan deviation will give a reduced noise in the low frequency range in frequency domain in Fig. 2(b) with^19^ *α* > 0.

**Figure 3:**
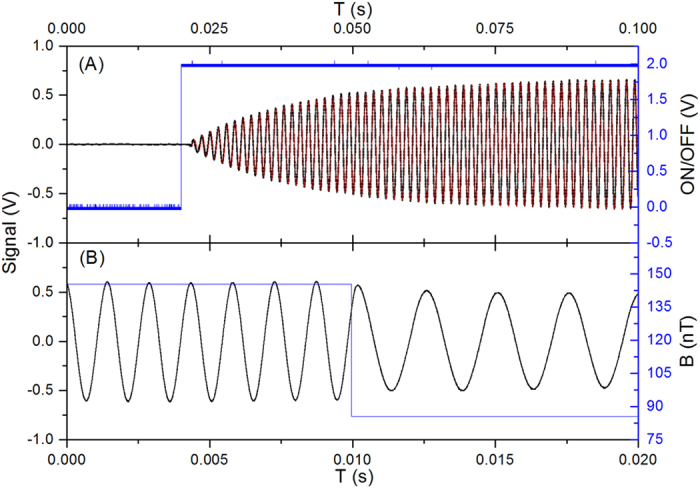
(**A**) Black line: magnetometer starts from the noise background, plotted against the upper time axis. The self-sustaining feedback loop is turned on at high level (blue line). The red line is fitted to estimate the starting time (20 ms). **(B)** Black line: the magnetometer’s response to a sudden magnetic field change of 60 nT, plotted against the bottom time axis. The change of the magnetic field is made within 1 μs (blue line).

Furthermore, we test the dynamic response of the magnetometer by applying sudden interruptions. This magnetometer can self-oscillate from the noise background without any initial pumping preparation and can regain self-oscillation after an interruption in the feedback loop in about 20 ms. Magnetometers of this type can quickly respond to sudden magnetic changes (60 nT within 1 μs, Fig. 3). Finally, we measure the Larmor frequency shift due to the circularly polarized pump light’s AC Stark effect^20^. The pulsed pump light leads to a much smaller effective intensity and reduced light shift. The Larmor frequency shift due to the pump light is as small as about 1.8 mHz/(mW/cm^2^), corresponding to about 380 f T/(mW/cm^2^) in the experiment.

In conclusion, we report a novel self-sustaining atomic spin magnetometer based on coherent optical pumping, demonstrate and investigate its features in details. As shown above, such a coherent pumping method can dramatically improve the performance of the magnetometer. The spin in this method behaves like a driven oscillator. The demonstrated 1/*τ* characteristics of noise level reduction via averaging in time using the self-sustaining method is very attractive and the method itself can be applied to other systems such as atomic clocks. The fast averaging, 1/*τ* rule enables magnetometers to reach the quantum shot noise floor of spin projection in a much shorter time. Also the magnetometer has a quick response to magnetic field variations and a reduced dependence on light shift. Since optical pumping can also be efficiently realized using spectral lamps, the magnetometer demonstrated here has a good prospect for miniaturization.

## Additional Information

**How to cite this article**: Xu, C. *et al*. A self-sustaining atomic magnetometer with *τ*^−1^ averaging property. *Sci. Rep.* **6**, 28169; doi: 10.1038/srep28169 (2016).
